# Therapeutic effects of lincomycin and level of drug degradation in broiler tissues after treatment

**DOI:** 10.14202/vetworld.2024.1026-1034

**Published:** 2024-05-09

**Authors:** Agustina Dwi Wijayanti, Alfian Yusak Muzaki, Cahyo Wibisono, Dyah Ayu Widiasih

**Affiliations:** 1 Department of Pharmacology, Faculty of Veterinary Medicine, Universitas Gadjah Mada, Yogyakarta, Indonesia; 2 Department of Veterinary Public Health, Universitas Gadjah Mada, Yogyakarta, Indonesia

**Keywords:** broiler tissues, lincomycin level, maximum residue limit, minimum inhibitory concentration

## Abstract

**Background and Aim:**

Lincomycin is an antibiotic used in broiler farming and is commonly combined with other substances to achieve synergistic and complementary effects on the antibacterial spectrum and mechanism. We developed a specific high-performance liquid chromatography (HPLC) method to measure lincomycin levels in broiler tissues. This study aimed to determine the lincomycin level in tissues and compare it with the minimum inhibitory concentration (MIC) and maximum residue limit (MRL) of certain pathogenic bacteria.

**Materials and Methods:**

Three groups of broiler chickens were involved in the study (n = 20 in each group): A control group without lincomycin treatment and two groups (each further divided into two sub-groups) that received oral lincomycin at a dose of 1 g/10 kg of body weight daily for 7 and 14 consecutive days. Tissue samples were collected from each group 1 day and 1 week after lincomycin administration (ALA). This study validated the development of a technique for analyzing drug level degradation in tissues using HPLC. Descriptive and statistical analyses were performed for drug levels to assess their therapeutic value and safety based on lincomycin MIC of certain pathogenic bacteria and MRL.

**Results:**

The method validation resulted in linear regression and coefficient of determination for tissues with r^2^ > 0.99, with a recovery rate of 90%–110%, precision as the coefficient of variation 15%, and specificity with no peak overlap for lincomycin. The limits of detection for the liver and kidney were 0.01 μg/g, 0.05 μg/g, and 0.1 μg/g for the breast muscle and all tissues. Administration of lincomycin for 7 and 14 days resulted in therapeutic value concentrations. Lincomycin levels in the liver and kidney of ALA exceeded the MRL, whereas breast muscles were below the MRL for a week of ALA treatment.

**Conclusion:**

Administration of lincomycin for 7 and 14 consecutive days resulted in therapeutic value; however, after a week, most tissues showed high drug concentrations that exceeded the MRL. It is necessary to carefully consider the prolonged therapeutic dose of lincomycin in broilers. Antibiotic therapy must be guided in such a way as to protect the product from harmful residues.

## Introduction

Lincomycin has long been used to treat animal infections in the United States and the European Union [1]. This antibiotic was no longer used due to widespread use of tetracyclines, aminoglycosides, and fluoroquinolones, which are considered more effective. However, due to the emergence of antimicrobial resistance problems in several classes of currently used antimicrobials, it is necessary to re-examine antibiotics that have not been used for a long time. The effectiveness of lincomycin and spectinomycin as beneficial therapies for bacterial infections in chickens has been studied [1, 2]. Lincomycin is derived from the actinomycete *Streptomyces lincolnensis* and has antibacterial activity against streptococci, pneumococci, and staphylococci. It belongs to the class of lincosamides and is typically categorized as macrolides, lincosamides, and streptogramins [1]. It prevents chronic respiratory disease (CRD) associated with *Mycoplasma* and chicken coliform infection [3, 4]. Moreover, the combination of lincomycin and spectinomycin has proven effective in inhibiting the growth of *Enterococcus cecorum* and influencing the fecal microbiota of broilers [4]. A previous study by Khan *et al*. [2] on the effectiveness or toxicity of serial doses of lincomycin-spectinomycin found it to be effective and safe at 20–100 mg/kg of body weight.

To measure the duration of drug efficacy and the safety of poultry products, it is essential to study drug degradation in chickens, including pharmacokinetic and residue studies. To ensure the safe consumption of the drug, it must be calculated below the maximum residue limit (MRL) in all edible tissues. Antimicrobials are used for both therapeutic and non-therapeutic purposes in farm animals. Despite regulations prohibiting the use of antimicrobials as growth promoters, violations have occurred; therefore, a control and screening method to measure antimicrobial levels in edible tissues is required. Kim *et al*. [5] showed that the residue of lincomycin in laying hen eggs was low, ranging from low to high doses that were orally or intravenously administered. Studies on the pharmacokinetics and concentration of lincomycin in poultry tissues have been conducted [6–8], suggesting that the use of lincomycin for poultry therapy is being reconsidered. After a prolonged period of disuse, lincomycin has re-emerged as a therapy for poultry in Indonesia, as reported in a study of the lincomycin-spectinomycin formulation [9]. The use of lincomycin along with spectinomycin was highest in Java Island, the central broiler farming region, compared with other areas in Indonesia. Lincomycin-spectinomycin is used to treat enterococcus infections that cause necrotic enteritis in chickens in Germany [4] and to combat CRD infection in China [2].

In addition to therapeutic purposes, research on lincomycin degradation in tissues after oral application in broilers is essential to evaluate the duration of drug efficacy and to analyze the safety of the resulting residue in the product. This study aimed to determine the therapeutic level of lincomycin by comparing it with the minimum inhibitory concentration (MIC) of certain pathogenic bacteria and measuring the levels of the MRLs as safety values in animal edible tissues.

## Materials and Methods

### Ethical approval

This study was approved by the Ethics Commission of the Faculty of Veterinary Medicine, Universitas Gadjah Mada (Approval letter number 00100/EC-FKH/Int./2021).

### Study period and location

This study was conducted from September 2021 to June 2022 at the Animals Research and Study Unit and Pharmacology Department at the Faculty of Veterinary Medicine, Universitas Gadjah Mada, Yogyakarta, Indonesia.

### Materials

Materials used in this research were lincomycin in oral formulation Interspectin-L WS® (PT Tekad Mandiri Citra, Bandung), lincomycin hydrochloride monohydrate Vetranal™, analytical standard product number 31727 (Sigma-Aldrich USA), Shimadzu 6.1 high-performance liquid chromatography (HPLC) equipment used with the control system SCL-10 A VP, CTO-10 AC VP oven, DGU-14A degasser (Shimadzu version 6.1, Tokyo, Japan), Shimpack C18 column with a size of 4.6 150 mm, day-old chicken broiler of Cobb Strain (PT Janu Putera Sejahtera, Gunung Kidul, Yogyakarta), BR 1 feed free antibiotic (PT Japfa Comfeed Indonesia Tbk.), postal cage, micropipette (Socorex, Acura 825, Swiss), vortex mixer (Barnstead), centrifuge (Hettich, Germany), Millipore filter, electric balance (Ohaus), volumetric flask, Baker glass, centrifuge tubes, and chemicals.

### Validation method for determining lincomycin level in tissues

Abualhasan *et al*. [10] developed a method to determine the drug level from the liver, kidney, and muscle. The technique was validated by considering that linearity, precision, accuracy, specificity, limit of detection (LOD), and limit of quantification (LOQ) were sufficient to fulfill the specified requirements. Drug concentrations in tissues were measured by HPLC analysis. Phosphate buffer was prepared by mixing 13.5 g of phosphoric acid with 600 mg of hexane-sulfonic acid sodium salt in 800-mL aquabidest. The phosphate buffer solution was mixed with acetonitrile (89%:11% volume ratio) to prepare the mobile phase. At a wavelength of 220 nm, a temperature of 35°C, a flow rate of 1 mL/min, an injection volume of 20 μL, and a running time of 15 min, the solution was detected. The standard analytical concentration of lincomycin was determined using 11 serial concentrations of the drug (100, 50, 10, 5, 1, 0.5, 0.1, 0.05, 0.001, 0.005, and 0.0005 μg/mL) and spiking the drug into blank tissues determined using 100, 50, and 1 μg/mL of standard analytical lincomycin concentrations to find the linear regression equation Y = bx + a (Y = ratio peak area; b = slope; x = concentration; a = intercept). As a preliminary study, a validation method to measure the serial concentrations of lincomycin was performed [9, 11].

### Field trial of lincomycin oral formulation (LoF) to broilers

A total of 60 Cobb strain broilers were reared from day-old chickens to 38 days old. The chickens were grouped into three groups (n = 20), namely, G0 as the control with no treatment of LoF, G1 given LoF during a week (7 days), and G2 given LoF during 2 weeks (14 days) with a dose of 0.75 g/L of drinking water or similar to 1 g/10 kg of body weight, daily. The chicken houses were built from hard and solid materials, with good air circulation and ventilation, a temperature of 29°C–32°C, a humidity of 75%–80%, and dry husk litter laid on dry litter. The chickens were *ad libitum* fed BR 1 commercial non-antibiotic feed and tap water. The flock also received vaccination against New Castle disease and infectious bursa disease on days 4 and 21, respectively. LoF treatments were applied from day 17 to day 22 (G1) and from day 17 to 29 (G2). Necropsy was performed after the chicken was slaughtered by the halal method, and organ (liver, kidney, and breast muscle) sampling was conducted on day 23 (G1.1), day 30 (G1.2 and G2.1), and day 38 (G2.2). Tissue samples were kept frozen at –23°C in the freezer until analysis.

### Extraction of liver, kidney, and breast muscle for lincomycin level measurements

The drug concentrations in the liver, kidney, and breast muscle were measured using HPLC analysis, which has been briefly validated. Chicken liver, kidney, and breast muscle tissues from broilers were extracted by mincing and homogenizing the tissues, weighing 1 g, and then placing them in a centrifuge tube. Next, 2.5 mL of 1% acetonitrile acid (1 mL anhydrous acetic acid was added in 100 mL acetonitrile), vortexed for 5 min, and centrifuged (3000× *g*) for 10 min. The supernatants were separated and evaporated in a water bath for 10 min until dry. After drying, 1.5 mL of buffer was added to phosphate (pH 7.4), 2 ml of N-hexane (pH 7.4), vortexed for 5 min, and centrifuged at 3000× *g* for 10 min. The supernatant was collected, N-hexane was discarded, and three repetitions were performed. The obtained supernatant was then centrifuged (2500× *g*) for 15 min, filtered with a 0.45 μm Millipore filter, and injected into HPLC at a volume as high as 20 μL.

### Therapeutic level measurement and drug residue analysis

The minimum inhibitory concentration (MIC) of lincomycin for some pathogenic bacteria in poultry (*Escherichia coli*, *Salmonella* Enteritidis, *Mycoplasma synoviae*, *Staphylococcus aureus*, and *Pasteurella multocida*) was used to measure the efficacy of LoF [12–18]. The drug residues were analyzed by comparing them with the MRLs of lincomycin according to the Indonesia National Standard [19]. The level of lincomycin among groups was statistically analyzed with a one-way analysis of variables using the Statistical Package for the Social Sciences v.16 (IBM Corp., NY, USA).

## Results

### Validation method for determining lincomycin concentration in broiler tissues

**Figure-1:**
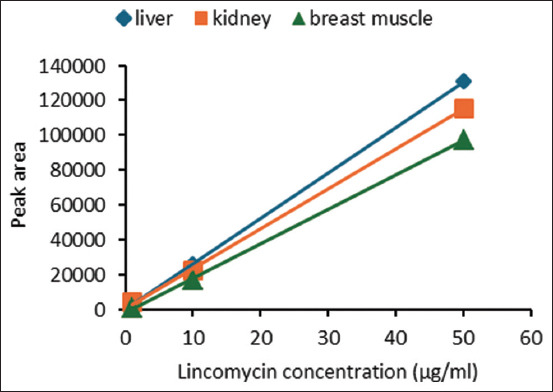
Linear regression for concentrations of lincomycin in tissues.

**Figure-2:**
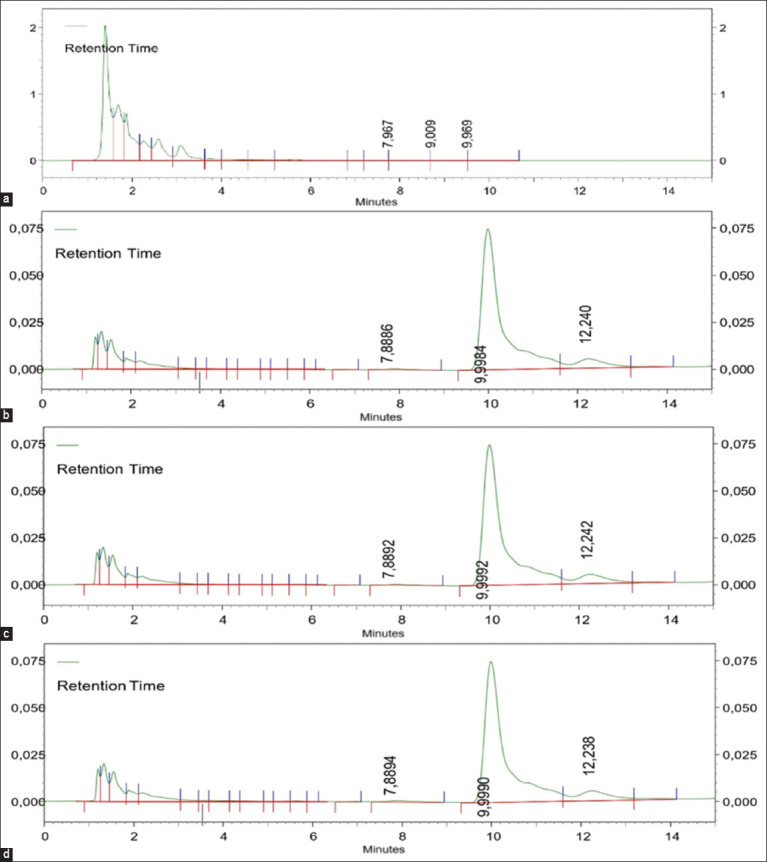
The peak area of lincomycin concentration is 100 µg/g appears at 9.9 min in spiking broiler tissues. (a) liver blank tissues, (b) liver, (c) kidney, and (d) breast muscle.

We validated a method for quantifying lincomycin from tissues using a preliminary approach. Table-1 describes the linearity, precision, accuracy, specificity, LOD, and quantification results for the liver, kidney, and breast muscles. Figures-1 and 2 show the linearity graph and validation results for determining lincomycin concentrations in broiler tissues.

**Table 1:** The validated method results for determining the lincomycin concentration in broiler tissues.

Values	Results
Linearity	The linear regression and coefficient determination
	y = 2607.7x + 254.33, r² = 0.9999 (liver)
	y = 2277.3x + 1777.7,
	r² = 0.9996 (kidney)
	y = 1980.9x - 1046.4, r² = 0.9996 (breast muscle)
Accuracy	Value of % recovery 90%–110%
Precision	Value of % coefficient of variation (CV) ≤15%
Specificity	There was no overlap peak for lincomycin. The peak area appears at min 9.9
LOD	0.01 μg/g (liver, kidney) and 0.05 μg/g (breast muscle)
LOQ	0.1 μg/g (liver, kidney and breast muscle)

LOD=Limit of detection, LOQ=Limit of quantification

The linearity of the calibration curve with a deviation value (r² > 0.99) represents the linear correlation between the drug concentration and peak area [20]. Determination of the precision value (% of coefficient variation) and accuracy in this study was performed through nine repetitions with concentrations of 50 (μg/g), 10 (μg/g), and 1 (μg/g) of lincomycin spiked to the blank tissues. Precision is considered good if it is <15% [21]. According to Maddaleno *et al*. [22], HPLC is a suitable method for detecting drug levels in broiler chicken tissue if it has a recovery value between 80% and 110%. The specific peak at 9.9 min indicates the specificity of lincomycin detection in tissues (Figure-2). The LOQ for all tissues is 0.1 μg/g, and the LOD for the liver and kidney is 0.01 μg/g and that for breast muscle is 0.05 μg/g, indicating sufficient levels to detect and measure lincomycin in broiler tissue.

### Levels of lincomycin in the liver, kidney, and breast muscle

Measurements of lincomycin levels in different groups revealed levels within 1 day and 1 week after lincomycin administration (ALA). The drug levels in the kidney and breast muscle experienced degradation from 1 day until a week of ALA with treatment durations of 7 or 14 days consecutively, with levels in breast muscle below the MRL after a week of ALA (Table-2) [19]. These results suggest that the therapeutic use of lincomycin in broilers should take up to 7 days to ensure the safety of the breast muscle. Drug levels in the liver decreased from 1 day to a week of ALA (from 2.44 ± 1.42 to 1.74 ± 0.73 μg/g) for the 7-day administration. However, at 14 days of administration, the level of the drug in the liver until a week of ALA was relatively unchanged (2.48 ± 1.12 μg/g in a day of ALA and 2.50 ± 1.10 μg/g in a week of ALA) and had no significantly different levels in the G1 and G2 groups.

**Table 2:** The lincomycin levels in broiler tissues after 7 and 14 days of consecutive oral administration. The drug level was detected on 1 day and 1 week ALA.

Group	Lincomycin level (µg/g)		

	Liver	Kidney	Breast muscle
G0	ND	ND	ND
G1.1	2.44 ± 1.42^a^	2.65 ± 1.38^a^	0.11 ± 0.08^a^
G1.2	1.74 ± 0.73^a^	1.30 ± 0.95^a^	0.08 ± 0.6^a^*
G2.1	2.48 ± 1.12^a^	0.90 ± 0.83^b^	0.68 ± 0.36^b^
G2.2	2.50 ± 1.10^a^	0.38 ± 0.28^b^	0.08 ± 0.06a*

G0 No lincomycin administration; G1.1 lincomycin per oral administration during 7 days, drug level detected 1 day of ALA; G1.2 lincomycin per oral administration during 7 days, drug level detected 1 week of ALA; G2.2 lincomycin per oral administration during 14 days, drug level detected 1 day of ALA; G2.2 lincomycin per oral administration during 14 days, drug level detected 1 week of ALA. ^a,b^The different symbols in the same column show a significant difference (p < 0.5),

* Below the maximum residue limit Standard Nasional Indonesia [19], ND=Not detectable, ALA: After lincomycin administration

## Discussion

### Lincomycin levels in broiler tissues after administration

The validated method was established to measure broiler lincomycin levels from liver, kidney, and breast muscles because it has good linearity, accuracy, precision, and specificity (Table-1). The LOD and LOQ values (0.01–0.05 μg/g and 0.1 μg/g) were good for measuring detectable and quantitative lincomycin residues in the tissue. Table-2 shows high levels of drugs in the liver and relatively higher levels of drugs in the kidney compared with those in the breast muscle. A normal liver has good perfusion, and the drug enters the hepatocytes very quickly and is metabolized. A study of liver metabolism showed a correlation between liver viscoelastic properties and metabolism function. Alterations in blood flow and drug interactions prompt adaptation of liver function [23].

A study of drug residues in farm animals has also revealed that the liver has a high drug content [24–26]. Therefore, in this study, high levels of lincomycin were retained in both liver and kidney for a week after administration. Therefore, lincomycin must be considered as a safe product to consume. The half-life of lincomycin after oral administration at a dose of 10 mg/kg of body weight in broilers is approximately 7 h [27]. For therapeutic purposes, multidose administration will cause the accumulation of drugs that may alter the elimination phase.

The duration of drug administration in broiler farms is important for both treatment and food safety. It is sometimes necessary to prolong the treatment duration to maintain the drug’s therapeutic level in the body and to ensure the effective elimination of infectious agents. Residue refers to any compound present in edible tissues that results from the use of a drug and includes the drug, its metabolites, and any other substance formed in or on food because of the drug’s use [28]. To ensure food safety for consumers, particularly when harvested, drug residues in edible tissues must be continuously monitored not to exceed the MRL. The presence of antimicrobial residues in meat, milk, egg, and fish is a serious public health threat due to the potential induction of antimicrobial resistance [29]. The involvement of veterinary drugs in the food chain creates a serious health problem; therefore, regulations governing the use of antimicrobials as therapy in veterinary medicine need to be monitored. In many countries, public health agencies or veterinary authorities have established regulatory tolerances for registered veterinary drugs [30]. To ensure the health of the consuming public, these regulations must determine the tolerance values for veterinary drug residues contained in animal foods.

### MIC of lincomycin and therapeutic levels in tissues

Lincomycin exhibits good bactericidal activity against Gram-positive bacteria by inhibiting protein synthesis [31]. Lincomycin is often combined with other antimicrobials, such as kitasamycin [32], spectinomycin [4, 32, 33], diclazuril [14], and bacitracin [15], to enhance its effectiveness. Clinically, lincomycin, either alone or in combination, is used to combat necrotic enteritis [15, 32], CRD, *S. aureus*, and *Mycoplasma* species infections [2]. The use of lincomycin as a therapeutic agent has been increasing tremendously because of its greater effectiveness against poultry disease and its low cost [16]. The use of lincomycin as a combination formulation has recently increased in Indonesia as a therapeutic agent in poultry. Muzaki *et al*. [9] used snowball sampling, and 202 respondents from 26 provinces in Indonesia reported that 11.8%–26.6% of lincomycin formulations were used as therapeutic agents in poultry farms on Java Island and 0.5%–6.9% were used in farms outside Java Island. Java Island is the center of poultry farming in Indonesia.

The minimum or maximum MIC values represent the responses of the antibacterial activity of the drug to different strains and isolate sources. When the highest antimicrobial concentration did not inhibit growth, the MIC value was greater than (>) that of the highest antibiotic concentration in the plate. On the other hand, if the lowest antibiotic concentration inhibited growth, the MIC value was expressed as lower than or equal (≤) to this concentration [17]. Drug levels in the liver and kidney were either the same or higher than the MIC for *M. synoviae*, *S. aureus*, and *Mycoplasma gallisepticum* [17] in treatment groups. The drug levels in the breast muscle of G2.1 (detected a day after 14 days of oral ALA) remained consistent with the MIC value of lincomycin for *M. synoviae*. Liver and kidney drug levels have therapeutic values for some pathogenic bacteria in poultry. There is a limit to the drug level in the breast muscle, which was observed only in G2.1, which possesses therapeutic value. The level of lincomycin in the liver has medicinal value in all groups, indicating the long-term persistence of the drug in the liver. Table-3 [12, 13, 17, 18, 34, 35] compares the drug levels in broiler tissues with the MIC values of lincomycin from recent studies. Several antimicrobial drugs, such as tetracycline [25], enrofloxacin [36], florfenicol [37], and tilmicosin [38], have been reported to have higher doses in the liver than in the muscle or kidney of broilers. The high MIC values of *E. coli* and *P. multocida* indicate that lincomycin resistance may have emerged from these bacteria (Table-3). Lincomycin resistance has been found in broilers, showing a zero inhibition zone against *Salmonella* Typhi, *S. aureus*, and *E. coli*, whereas tetracycline had a more expansive inhibition zone [39]. In contrast, lincomycin showed a lower number of resistance genes (*lnu*A) than tetracyclines (*tet*A, *tet*B, *tet*M) and aminoglycosides [40].

**Table 3:** The MIC values of lincomycin for some pathogenic bacteria compared to the lincomycin levels found in tissues of broiler after oral administration for 7 or 14 days consecutively.

MIC of lincomycin (µg/mL)		Microbial	Drug level in tissues exceeds or is the same as MIC	References

Minimum	Maximum			
1 62.5		*Staphylococcus aureus*	G1.1 and G1.2 (liver, kidney); G2.1 and G2.2 (liver)	[12, 13]
512, 125		*Escherichia coli*	(The drug in all tissues below MIC)	[12, 13]
512, 62.5		*Pasteurella multocida*	(The drug in all tissues below MIC)	[12, 13]
≤0.5 0.5	> 32	*Mycoplasma sinoviae*	G1.1; (liver, kidney); G1.2 (liver, kidney); G2.1 (liver, kidney, breast muscle); G2.2 (liver, kidney)	[17, 18]
1	11	*Mycoplasma gallisepticum*	G1.1 and G1.2 (liver, kidney); G2.1 and G2.2 (liver)	[34]
1	4		G1.1 and G1.2 (liver, kidney); G2.1 and G2.2 (liver)	[35]

G1.1 lincomycin per oral administration during 7 days, drug level detected 1 day of ALA; G1.2 lincomycin per oral administration during 7 days, drug level detected 1 week of ALA; G2.2 lincomycin per oral administration during 14 days, drug level detected 1 day of ALA; G2.2 lincomycin per oral administration during 14 days, drug level detected 1 week of ALA, ALA=After lincomycin administration, MIC=Minimum inhibitory concentration

In particular, the avian pathogenic *E. coli* (APEC) strain, which causes colibacillosis, causes high morbidity and mortality of poultry worldwide. APEC resistance to ampicillin, amoxicillin, ceftiofur, streptomycin, gentamicin, kanamycin, chloramphenicol, florfenicol, tetracycline, co-trimoxazole, nalidixic acid, ciprofloxacin, and enrofloxacin has been reported [41]. In addition, data on antibiotic resistance of *P. multocida* in poultry, such as ampicillin, gentamicin, erythromycin, tetracycline, co-trimoxazole, and enrofloxacin, have been reported. This study revealed that lincomycin, along with chloramphenicol, ciprofloxacin, norfloxacin, enrofloxacin, and gentamicin, had low levels of resistance; it was observed in 10% of isolates, thus remaining the only effective therapeutic alternative [38]. Similar to an antimicrobial resistance study [42], lincomycin was found to be effective against *Streptococcus agalactiae* isolates in combination with 16 other antibiotics.

### Lincomycin levels in broiler tissue as a consideration of permissible residue limits

**Figure-3:**
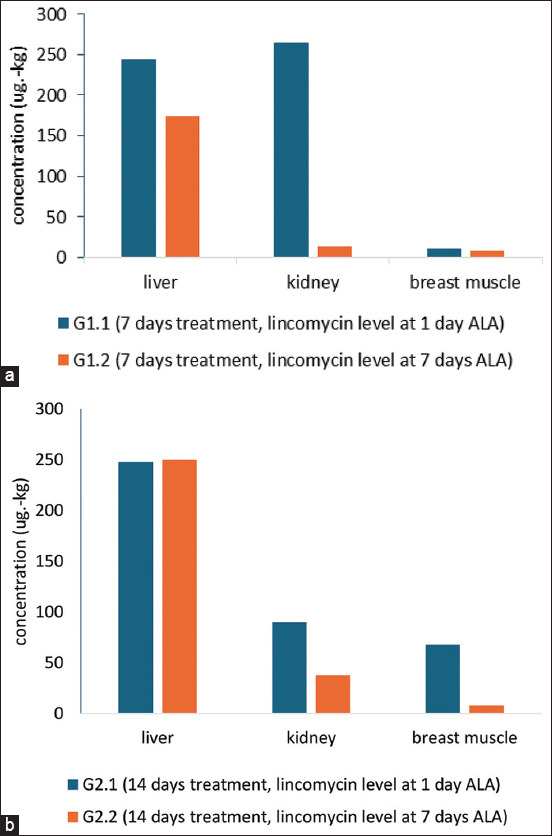
The degradation level of lincomycin in tissues; (a) 7 days of treatment, with lincomycin levels at 1 and 7 days of ALA and (b) 14 days of treatment, with lincomycin levels at 1 and 7 days of ALA (ALA=After lincomycin administration).

Lincomycin levels in broiler breast muscle after 7 and 14 consecutive days of oral administration were 0.08 ± 0.06 μg/g, which is below the MRL of the Indonesian Standard for lincomycin residue in edible tissues of 0.1 μg/g [19]. However, liver and kidney lincomycin levels were still above the MRL (Table-2). After long-term treatment, drug residues are often found for a longer period and at higher concentrations in edible tissues. To ensure the food safety of animal products intended for consumption, the drug residue limit shall be determined by the commission or authorization. Codex Alimentarius [43] has set a limit of 0.2 μg/g for muscle tissue, 0.5 μg/g for liver tissue, 0.1 μg/g for fat tissue, and 0.5 μg/g and 1.5 μg/g for kidney tissue of poultry and swine. Meanwhile, the European Commission [44] has set an MRL for lincomycin in any farm animal species as follows: 0.1 μg/g for muscle tissue, 0.05 μg/g for fat tissue and eggs, 0.5 μg/g for liver tissue, and 1.5 μg/g for kidney tissue, whereas for cattle milk, the MRL was 0.15 μg/g [43, 44]. Park *et al*. [45] conducted a study of lincomycin levels in broiler tissues and found that after 5 days of the orally recommended dosage, the drug rapidly decreased within 3 days. The withdrawal time (WT) was determined based on the duration of pharmacokinetic breaks and linear regression analysis while considering the MRL value. Degradation of the drug has a range of velocities depending on the form of the drug used. Figure-3a showed the significant level degradation of 7 days consecutive lincomycin treatment, at 1 day and 7 days ALA in the liver and kidney, but slow degradation at breast muscle. Figure-3b describes lincomycin level degradation in broiler tissues with a unique concentration in the liver after 14 days of treatment, that drug level in the liver had no degradation

The therapeutic management of broilers must be consistent in producing food products of animal origin that are safe for consumption. The use of antibiotics is still high in broilers involved in farm management [46]. Therefore, the selection of therapeutic agents, doses, and duration of use must take into account drug levels that do not exceed the established safe residue limit (MRL). A study conducted by Schreier *et al*. [4] revealed that lincomycin formulation given to broilers infected and non-infected with *E. cecorum* inhibited bacterial growth until 3 weeks after the last administration of the drug. This may be due to the therapeutic level of the drug maintained for 3 weeks after administration for 6 days. WT lincomycin typically ranges from 1 to 5 days for broilers’ lincomycin formulation [44, 47, 48]. However, the duration of treatment should be taken into account because lincomycin may extend the duration of treatment in the tissues.

## Conclusion

The duration of lincomycin administration for both 7 and 14 consecutive days in broilers resulted in therapeutic value. However, after a week of ALA, drug degradation in most tissues showed high concentrations that exceeded the MRL limit. Long-term therapeutic doses of lincomycin in broilers should be carefully considered, as this may lead to drug concentrations that exceed the safe residue limit in broiler tissues. The use of antibiotics for the treatment of diseases in broilers must be guided in such a way as to ensure the safety of the product from harmful residues.

## Authors’ Contributions

ADW: Drafted the manuscript, designed the method, and constructed the results and discussion. AYM and CW: HPLC analysis, validated method, field trial, and samplings. DAW: Results analysis and reviewed the manuscript. All authors have read, reviewed, and approved the final manuscript.
